# Uric acid level in climacteric women and its association with clinical and metabolic parameters

**DOI:** 10.1038/s41598-023-35287-1

**Published:** 2023-05-25

**Authors:** Laura Alves Cota e Souza, Georgia Carvalho de Oliveira D’Angelo, Glenda Nicioli da Silva, Angélica Alves Lima

**Affiliations:** 1 Programa de Pós-Graduação em Ciências Farmacêuticas (CiPharma), Escola de Farmácia, Universidade Federal de Ouro Preto, Morro do Cruzeiro, S/N, Ouro Preto, MG CEP 35400–000 Brazil; 2 Departamento de Análises Clínicas (DEACL), Escola de Farmácia, Universidade Federal de Ouro Preto, Ouro Preto, Brazil

**Keywords:** Biomarkers, Health care, Risk factors

## Abstract

Climacteric women often experience unfavorable metabolic changes. Consequently, identifying markers that may contribute to such undesirable changes is imperative. This study aimed to evaluate serum uric acid (UA) concentration and its association with metabolic and clinical parameters in climacteric women. We selected 672 women between 40 and 65 years and performed interviews, biochemical analyses, blood pressure, and anthropometric measurements. UA levels were determined using the enzymatic-colorimetric method. We compared variables according to the quartiles of UA using the Kruskal–Wallis test. The mean UA level was 4.9 ± 1.5 mg/dl, ranging from 2.0 to 11.6 mg/dl. We found that UA levels greater than 4.8 mg/dl were associated with adverse metabolic parameters in climacteric women. For all anthropometric and biochemical variables, we observed significantly better results in women who had lower UA levels (p < 0.05). Similarly, we observed a significant increase in blood pressure, frequency of metabolic syndrome, and cardiovascular risk as UA levels increased (p < 0.05). Our findings showed that climacteric women with high levels of UA were more likely to have adverse metabolic and clinical parameters than those with lower UA levels. Further studies may determine the causal relationship between UA and metabolic changes in climacteric women.

## Introduction

The aging process in women is characterized by a decline in serum estrogen concentrations, which significantly increases the risk of cardiovascular and metabolic diseases^1^. Studies have shown that women experience an increase in weight, body fat percentage, and waist circumference during the menopausal transition and postmenopause^1,2^. The lipid profile of postmenopausal women becomes more atherogenic, with elevated levels of total cholesterol, LDL cholesterol, and apolipoprotein B, irrespective of age, ethnicity, or weight^1,3^.

Several studies have established a link between uric acid (UA) and an increased risk of metabolic disorders associated with menopause^4–6^. UA is a metabolite derived from purine oxidation, which occurs when nucleotides break down^7^. The serum concentration of UA remain relatively constant due to a balance between its production and excretion. However, conditions that increase UA production or decrease UA excretion can lead to hyperuricemia, which is generally defined as a UA blood concentration greater than 7.0 in men and 6.0 mg/dl in women^7,8^.

Hyperuricemia can be caused by many factors, including changes in purine metabolism, high protein intake, enzyme dysfunction, exposure to xenobiotics, and kidney or liver disease. Certain life stages, such as the third trimester of pregnancy and menopause, are also related to increased UA levels^9,10^.

Elevated levels of UA in the blood are known to cause the deposition of urate crystals in the joints and kidneys, increasing the risk of gout, renal disease, and hypertension. In recent decades, evidence has emerged demonstrating the clinical significance of UA beyond rheumatology. Numerous studies have linked UA to various metabolic disorders, including cardiovascular disease, metabolic syndrome, dyslipidemia, non-alcoholic fatty liver disease, and obesity^11^.

Although many studies have shown an association between hyperuricemia and metabolic diseases, few have examined the role of UA in menopausal changes. It is therefore important to investigate UA as a biological marker that may contribute to the development of metabolic diseases, leading to increased morbidity and mortality among middle-aged women. This study aimed to evaluate serum UA concentrations in climacteric women and assess their association with clinical and metabolic parameters.

## Methods

### Study design

This cross-sectional study evaluated women between 40 and 65 years. Participants were recruited through active search in the Basic Health Units of Ouro Preto, Minas Gerais, Brazil, using a non-probabilistic convenience sampling method. The exclusion criteria were gout diagnosis and the use of urate-lowering therapy.

Volunteers were invited to participate in the study by doctors, nurses, health agents, or researchers. All participants attended on predetermined days for an interview, blood collection, and anthropometric measurements. The study adhered to relevant guidelines and regulations, and all methods were carried out in accordance with them. The Research Ethics Committee of the Universidade Federal de Ouro Preto approved this study (Number: 56312916.8.0000.5150), and all eligible participants gave informed consent.

### Interviews

During the interviews, the women provided information on several aspects, including their age, level of education, marital status, number of children, income, personal and family history of diseases, tobacco and alcohol use, physical activity levels, medication use, age at menarche, sexual activity, date of their last menstrual period, any changes in their menstrual cycle, surgeries they may have had, years since menopause, and age at menopause.

We considered any medication used by the participants and classified them into the following categories: antihypertensive, antidepressant/anxiolytic, thyroid agents, lipid-lowering agents, vitamins/supplements, antidiabetic, hormones, analgesic/anti-inflammatory, and others.

Participants were classified into the late reproductive phase, menopausal transition, and postmenopause using the STRAW + 10 criteria. The primary criterion used was the menstrual pattern, while FSH levels and age served as supportive criteria^12^.

### Anthropometric and blood pressure assessments

We evaluated weight, height, body fat percentage (BF), and waist circumference (WC). We measured height using a simple portable stadiometer with an accuracy of 0.1 cm. Participants were instructed to stand with their arms along the body, feet together, and pointed forward. We assessed weight and body fat percentage using a Tanita® bioimpedance scale (Model 2204), with a graduation of 100 g. Participants stood upright, barefoot, and looked straight ahead during the measurements. We measured WC using a measuring tape at the midpoint between the last rib and the iliac crest. Using the anthropometric measurements, we calculated the body mass index ((BMI = weight (kg)/height (m)^2^) and waist-to-height ratio (WHtR = waist circumference (cm)/height (cm)).

We used a Bioland®—3005 digital wrist device to measure blood pressure. Participants were seated, with their feet on the floor and their left arm at heart level, following the manufacturer's recommended technique.

### Biochemical assessment

After 12 to 14 h of fasting, we collected blood samples from the participants. They were instructed to avoid intense physical exercise 24 h before blood collection and alcohol consumption for 72 h. We determined uric acid (UA), fasting blood glucose (FBG), insulin, lipid profile (triglycerides, total cholesterol, HDL, LDL), urea, and creatinine levels using the enzymatic-colorimetric method. HDL and LDL were analyzed using the direct homogeneous method, and hs-CRP was measured using the immunoturbidimetric method. We performed all biochemical analyses using the Cobas Integra® 400 Plus (Roche). We used the Access 2 Immunoassay System® (Beckman & Coulter) and chemiluminescence to determine insulin levels. All these determinations were performed in the Clinical Analysis Laboratory of the Federal University of Ouro Preto (LAPAC/UFOP).

We calculated the following indices: homeostasis model assessment of insulin resistance (HOMA-IR = FBG [mg/dl] × 0.0555 × insulin [µUI/l] 22.5^13^; non-HDL (TC [mg/dl] – HDL [mg/dl]); visceral adiposity index (VAI = [WC/(36.58 + (1.89 × BMI))] × [TG/0.81] × [1.52 /HDL])^14^; lipid accumulation product (LAP = [WC − 58] × TG [mmol/l])^15^; and atherogenic index of plasma (AIP = log TG [mmol/l]/HDL [mmol/l])^16^. We then classified women according to their AIP levels into groups of low cardiovascular risk (AIP < 0.11) and moderate/high risk (AIP ≥ 0.11)^17^.

We defined metabolic syndrome according to the Joint Interim Statement (JIS) harmonized criteria^18^. Participants who had three or more of the following alterations were considered as carriers of metabolic syndrome: WC ≥ 80 cm, triglycerides ≥ 150 mg/dl, HDL < 50 mg/dl, PAS ≥ 130 mmHg or PAD ≥ 85 mmHg or the use of antihypertensive medication, and FBG ≥ 100 mg/dl or the use of hypoglycemic agents.

### Statistical analysis

EpiData software (version 3.2) was used to double enter and check the data. The statistical analyses were performed using the Solution for Statistical Products and Services software (IBM SPSS 20.0). We used Pearson's chi-square test to compare categorical variables. We tested the distribution of quantitative variables using the Kolmogorov–Smirnov test and the Kruskal–Wallis test, followed by the Bonferroni correction, to compare the quantitative variables. For all statistical analyses, we considered a significance level of 5% (p < 0.05).

## Results

We initially selected 691 women aged between 40 and 65 years. However, we excluded 19 participants who reported a gout diagnosis or urate-lowering therapies, such as allopurinol and probenecid. Therefore, our study analyzed 672 participants.

**Figure 1:**
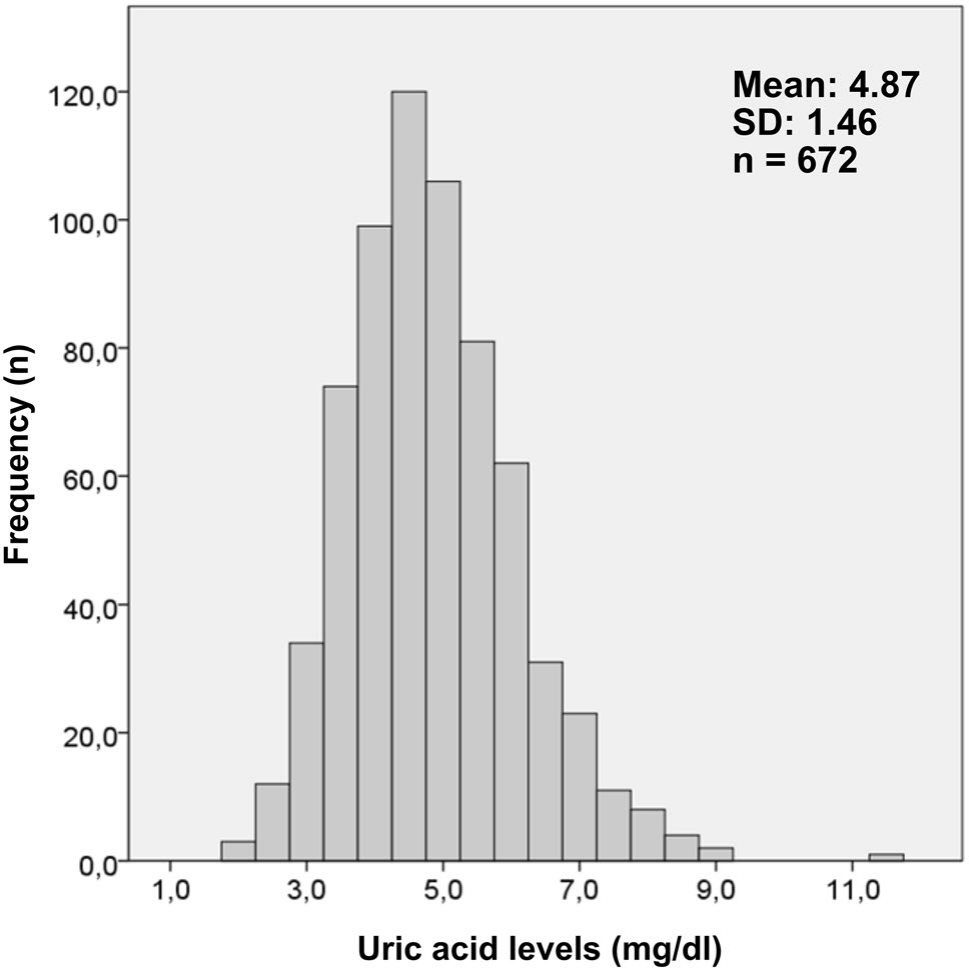
Levels of uric acid in the participants.

UA levels of the participants ranged from 2.0 to 11.6 mg/dl, with a mean value of 4.9 ± 1.5 mg/dl (Fig. 1). Among the women we studied, 14.9% (n = 100) had UA levels greater than 6.0 mg/dl, indicating hyperuricemia.

We analyzed the data by dividing participants into quartiles of UA. The first quartile (Q1) included women with UA levels up to 4.0 mg/dl, the second quartile (Q2) included women with UA levels between 4.1 and 4.8 mg/dl, the third quartile (Q3) included women with UA levels between 4.9 and 5.6 mg/dl; and the fourth quartile (Q4) women with UA levels greater than 5.6 mg/dl.

The median age of participants was between 50 and 53 years old (p = 0.002). In all quartiles of UA, most participants studied for more than 8 years, lived with a partner, used the public health system, did not smoke, did not have regular use of alcohol, and were sexually active and postmenopausal. In this study, we also included women after hysterectomy (10.4%, n = 70) since we found no significant differences between them and those who reported natural menopause for all parameters evaluated (data not shown). The frequency of postmenopausal women increased with higher UA quartiles (p = 0.007). In the first quartile, most of the participants were physically active (56.9%; n = 95), while in the fourth quartile, most of the women were sedentary (60.8%; n = 96) (p < 0.05). The use of drugs and hypertension frequency increased proportionally to UA levels. The statistical analysis showed that women in Q1 and Q2 used significantly lower medications than those in Q3 and Q4 (p < 0.001). Women in the fourth UA quartile had a significantly higher frequency of hypertension (58.2%; n = 92) than those in the first (18.6%; n = 31), second (27.4%; n = 48), and third quartiles (36.6%; n = 63) (Table 1).

**Table 1:** Sociodemographic and behavioral variables of the participants according to serum concentrations of uric acid.

Variable	Quartile of UA				p	χ^2^
	Q1	Q2	Q3	Q4		
	≤ 4 mg/dl	4.1–4.8 mg/dl	4.9–5.6 mg/dl	≥ 5.7 mg/dl		
	(n = 167)	(n = 175)	(n = 172)	(n = 158)		
	n (%) or median (1°–3° quartile)					
Age	50 (45–56)	51 (46–56)	51.5 (47–55)	53 (49–57)^ac^	**0.002**	14.480
Education						
0 to 8 years	48 (28.7)	54 (30.9)	70 (40.7)	68 (43.0)	**0.012**	10.885
More than 8 years	119 (71.3)	121 (69.1)	102 (59.3)	90 (57.0)^a^		
Marital status						
No partner	61 (36.5)	63 (36.0)	63 (36.6)	55 (34.8)	0.986	0.147
With partner	106 (63.5)	112 (64.0)	109 (63.4)	103 (65.2)		
Health system						
Public	97 (58.1)	102 (58.3)	100 (58.1)	105 (66.5)	0.081	11.231
Private	35 (21.0)	33 (18.9)	23 (13.4)	16 (10.1)		
Both	35 (21.0)	40 (22.9)	49 (28.5)	37 (23.4)		
Current smoker						
Yes	16 (9.6)	14 (8.0)	22 (12.8)	21 (13.3)	0.336	3.387
No	151 (90.4)	161 (92)	150 (87.2)	137 (86.7)		
Alcohol use^1^						
Yes	6 (3.6)	5 (2.9)	15 (8.7)	7 (4.4)	0.054	7.627
No	161 (96.4)	170 (97.1)	157 (91.3)	151 (95.6)		
Physical activity^2^						
Yes	95 (56.9)	81 (46.3)	86 (50.0)	62 (39.2)^a^	**0.014**	10.606
No	72 (43.1)	94 (53.7)	86 (50.0)	96 (60.8)		
Sexual activity						
Yes	119 (71.3)	135 (77.1)	129 (75.0)	108 (68.4)	0.276	3.865
No	48 (28.7)	40 (22.9)	43 (25.0)	50 (31.6)		
Stages of reproductive aging						
Reproductive stage	61 (36.5)	51 (29.1)	49 (28.5)	27 (17.1)	**0.007**	17.540
Menopausal transition	33 (19.8)	40 (22.9)	31 (18.0)	35 (22.2)		
Postmenopause	73 (43.7)	84 (48.0)	92 (53.5)	96 (60.8)^a^		
Use of drugs	97 (58.1)	104 (59.4)	127 (73.8)^ab^	126 (79.7)^ab^	** < 0.001**	25.921
Antihypertensive	40 (24.0)	53 (30.3)	58 (33.7)	71 (44.9)^ab^	**0.001**	16.979
Antidepressant/anxiolytic	34 (20.4)	29 (16.6)	36 (20.9)	39 (24.7)	0.338	3.371
Thyroid agents	19 (11.4)	25 (14.3)	18 (10.5)	16 (10.1)	0.617	1.789
Lipid-lowering agents	16 (9.6)	15 (8.6)	25 (14.5)	26 (16.5)	0.136	9.737
Vitamins/supplements	16 (9.6)	16 (9.1)	18 (10.5)	12 (7.6)	0.839	0.844
Antidiabetic	10 (6.0)	8 (4.6)	11 (6.4)	20 (12.0)^b^	**0.048**	7.904
Hormones	10 (6.0)	11 (6.3)	11 (6.4)	7 (4.4)	0.864	0.740
Analgesic/anti-inflammatory	6 (3.5)	5 (2.9)	9 (5.2)	10 (6.3)	0.410	2.884
Others^3^	17 (10.2)	17 (9.7)	22 (12.8)	21 (13.3)	0.907	1.618
History of disease^4^						
Neuropsychiatric disease	50 (29.9)	43 (24.6)	62 (36)	54 (34.2)	0.100	6.248
Hypertension	31 (18.6)	48 (27.4)	63 (36.6)^a^	92 (58.2)^abc^	**0.000**	62.051
Gastritis/cholecystitis	33 (19.8)	44 (25.1)	45 (26.2)	32 (20.3)	0.377	3.093
Thyroid disorders	21 (12.6)	29 (16.6)	23 (13.4)	27 (17.1)	0.570	2.013
Diabetes	12 (7.2)	10 (5.7)	15 (8.7)	26 (16.5)^b^	**0.004**	13.113

Significant values are in bold.

^a^p < 0.05 compared to Q1; ^b^p < 0.05 compared to Q2; ^c^p < 0.05 compared to Q3; ^1^more than or equal to 4 times a week; ^2^more than or equal to 3 times a week for at least 30 min; ^3^antiglaucoma agents, bisphosphonates, herbal remedies, bronchodilators, calcium channel blockers, appetite suppressants, antihistamines, proton pump inhibitors, muscle relaxants, antipsychotics, antiplatelet agents, and antivertigo agents; ^4^based on the woman's report of a previous doctor's diagnosis.

Table 2 shows the results of the anthropometric and biochemical data of the participants. Progressive median values were observed for all variables evaluated in this study according to UA levels. This indicates that women with lower UA levels had better results than participants with higher levels.

**Table 2:** Anthropometric and biochemical data of the participants according to serum concentrations of uric acid.

Variable	Quartile of UA				p	χ^2^
	Q1	Q2	Q3	Q4		
	≤ 4 mg/dl	4.1–4.8 mg/dl	4.9–5.6 mg/dl	≥ 5.7 mg/dl		
	(n = 167)	(n = 175)	(n = 172)	(n = 158)		
	Median (1°–3° quartile)					
Anthropometric variables						
WC (cm)	83.0	88.0	94.0	98.0	**< 0.001**	96.966
	(78.0–92.0)	(82.0–96.0)^a^	(85.0–101.0)^ab^	(89.0–106.0)^ab^		
WHtR	0.53	0.55	0.59	0.62	** < 0.001**	99.873
	(0.48–0.58)	(0.51–0.61)^a^	(0.53–0.64)^ab^	(0.57–0.67)^abc^		
Weight (kg)	61.2	66.6	71.7	73.0	** < 0.001**	81.775
	(54.6–68.6)	(59.0–75.6)^a^	(62.3–81.2)^ab^	(64.20–84.7)^ab^		
BMI (kg/m^2^)	24.4	26.2	27.9	29.5	**< 0.001**	101.830
	(22.1–27.1)	(23.0–28.8)^a^	(25.0–31.2)^ab^	(26.0–34.1)^abc^		
BF (%)	32.0	35.0	38.0	39.0	**< 0.001**	77.082
	(27.0–37.0)	(29.0–40.0)^a^	(32.0–42.0)^ab^	(34.8–44.0)^ab^		
Biochemical variables						
Fasting blood glucose (mg/dl)	89.0	90.0	92.5	97.5	**< 0.001**	37.800
	(82.0–97.0)	(84.0–97.0)	(87.0–102.8)^ab^	(88.0–109.3)^ab^		
Insulin (μUI/mL)	5.03	5.66	7.45	9.04	** < 0.001**	81.201
	(3.61–7.10)	(3.00–8.51)	(4.80–11.64)^ab^	(5.85–13.5)^ab^		
HOMA-IR	1.09	1.26	1.69	2.17	** < 0.001**	85.782
	(0.78–1.65)	(0. 83–1.92)	(1.12–2.82)^ab^	(1.36–3.33)^ab^		
Triglycerides (mg/dl)	87.0	98.0	112.5	147.5	** < 0.001**	86.454
	(66.0–115.0)	(74.0–135.0)	(84.0–161.3)^ab^	(111.8–195.3)^abc^		
TC (mg/dl)	202.0	206.0	208.0	218.0	**0.007**	12.216
	(180.0–227.0)	(180.0–237.0)	(184.5–233.0)	(188.0–252.5)^a^		
HDL (mg/dl)	60.0	56.0	54.0	50.0	** < 0.001**	37.456
	(50.0–73.0)	(47.0–68.0)	(45.0–65.0)^a^	(42.0–61.35)^abc^		
Non-HDL (mg/dl)	139.0	151.0	152.0	166.0	**< 0.001**	34.887
	(118.0–164.0)	(118.0–179.0)	(126.3–177.8)^a^	(136.8–201.5)^abc^		
LDL (mg/dl)	118.2	122.7	123.7	129.0	0.075	6.918
	(95.5–140.5)	(97.0–150.3)	(99.0–142.1)	(98.6–161.5)		
Urea (mg/dl)	27.0	28.0	28.0	30.0	**< 0.001**	26.036
	(23.0–31.0)	(23.0–32.0)	(24.0–34.0)	(26.0–35.0)^ab^		
Creatinine (mg/dl)	0.70	0.70	0.71	0.74	** < 0.001**	25.522
	(0.60–0.79)	(0.60–0.79)	(0.66–0.80)^ab^	(0.70–0.80)^ab^		

Significant values are in bold.

^a^p < 0.05 compared to Q1; ^b^p < 0.05 compared to Q2; ^c^p < 0.05 compared to Q3.

*WC* waist circumference, *WHtR* waist-to-height ratio, *BMI* body mass index, *BF* body fat, *HOMA-IR* Homeostasis model assessment of insulin resistance, *TC* total cholesterol, *HDL* high-density lipoprotein, *LDL* low-density lipoprotein.

The analysis of anthropometric variables showed that women classified in Q1 had significantly lower median values of WC, WHtR, weight, BMI, and percentage of BF than women allocated in Q2, Q3, and Q4 (p < 0.001). Similarly, women classified in Q2 had significantly better anthropometric results than volunteers in Q3 and Q4. Additionally, significant differences were found between Q3 and Q4 for BMI and WHtR (p < 0.001) (Table 2).

Biochemical variables analysis (Table 2) showed that women in Q1 and Q2 had significantly lower median values of FBG, insulin, HOMA-IR, TG, and creatinine compared to Q3 and Q4 (p < 0,050). Q1 and Q4 also differed significantly for total cholesterol (202 mg/dl vs. 218 mg/dl; p = 0.006), and urea (27 mg/dl vs. 30 mg/dl; p < 0.001). Participants in Q4 had lower levels of HDL (50 mg/dl) and higher levels of non-HDL (166 mg/dl) than those in Q1 (60 mg/dl and 139 mg/dl), Q2 (56 mg/dl and 151 mg/dl), and Q3 (54 mg/dl and 152 mg/dl) (p < 0.05). Although no significant differences were found for LDL, higher median values were observed with increasing levels of UA (p = 0.075).

We investigated the frequency and risk of metabolic syndrome among volunteers based on their levels of UA (Table 3). Women in Q4 had a significantly higher frequency of metabolic syndrome (64.4%; n = 102; p < 0.001) compared to participants in Q1 (22.8%; n = 38), Q2 (30.9%; n = 54), and Q3 (43.0%; n = 74). Moreover, we found a significant difference between Q1 and Q3 (22.8% vs. 43%; p < 0.001). We also noted a progressive increase in the risk of metabolic syndrome with increasing UA levels. Women in Q4 had an OR of 6.18 (95% CI 3.80–10.06; p < 0.001), while participants in Q3 and Q2 had an OR of 2.56 (95% CI 1.60–4.11; p < 0.001) and 1.52 (95% CI 0.93–2.46; p = 0.092), respectively.

**Table 3:** Frequency of metabolic syndrome and cardiovascular risk of the participants according to serum concentrations of uric acid.

Variable	Quartile of UA				p	χ^2^
	Q1	Q2	Q3	Q4		
	≤ 4 mg/dl	4.1–4.8 mg/dl	4.9–5.6 mg/dl	≥ 5.7 mg/dl		
	(n = 167)	(n = 175)	(n = 172)	(n = 158)		
Metabolic syndrome						
Frequency, n (%)	38 (22.8)	54 (30.9)	74 (43.0)^a^	102 (64.6)^abc^	**< 0.001**	67.208
Risk, OR (95% CI)	1	1.52 (0.93–2.46)	2.56 (1.60–4.11)^a^	6.18 (3.80–10.06)^a^	–	–
Cardiovascular risk						
SBP (mmHg), median (1°–3° quartile)	126.0	127.0	130.0	133.0	**0.001**	17.062
	(119.0–140.0)	(118.0–138.0)	(120.0–142.8)	(121.8–148.0)^ab^		
DBP (mmHg), median (1°–3° quartile)	80.0	82.0	82.5	85.0	**0.002**	14.886
	(74.0–87.0)	(75.0–89.0)	(76.3–90.0)	(78.0–92.0)^a^		
hs-CRP (mg/L), median (1°–3° quartile)	1.22	1.73	2.32	3.81	**< 0.001**	87.674
	(0.54–2.27)	(0.96–3.60)^a^	(1.20–4.67)^a^	(1.77–7.14)^abc^		
VAI, median (1°–3° quartile)	0.07	0.12	0.2	0.35	**< 0.001**	99.307
	(0.04–0.17)	(0.04–0.30)^a^	(0.08–0.49)^ab^	(0.17–0.81)^abc^		
LAP, median (1°–3° quartile)	25.34	32.4	44.07	61.95	**< 0.001**	87.329
	(15.58–39.81)	(20.62–51.30)^a^	(28.82–71.44)^ab^	(41.11–100.37)^abc^		
AIP, n (%)						
Low risk	145 (86.8)	144 (82.3)	113 (65.7)	80 (50.6)	< 0.001**< 0.001**	66.145
Moderate/high risk	22 (13.2)	31 (17.7)	59 (34.3)^ab^	78 (49.4)^abc^		

Significant values are in bold.

^a^p < 0.05 compared to Q1; ^b^p < 0.05 compared to Q2; ^c^p < 0.05 compared to Q3.

*SBP* systolic blood pressure, *DBP* diastolic blood pressure, *hs-CRP* High sensitivity C-reactive protein, *VAI* visceral adiposity index, *LAP* lipid accumulation product, *AIP* atherogenic index of plasma.

We also evaluated cardiovascular risk using the median values of systolic and diastolic blood pressure, hs-CRP, VAI, LAP, and AIP (Table 3). We observed significant differences between Q1 and Q4 for both systolic (126 mmHg vs. 133 mmHg; p = 0.002) and diastolic blood pressures (80 mmHg vs. 85 mmHg; p = 0.002). Furthermore, we noted a difference between Q2 and Q4 for systolic blood pressure (127 mmHg vs. 133 mmHg; p = 0.004). In terms of hs-CRP, VAI, and LAP, we observed a significant increase in these markers from the first quartile of UA to the fourth. The statistical analysis revealed differences in the comparison between all quartiles (p < 0.05) (Table 3). For AIP, we found that women in Q1 had a lower frequency of moderate or high cardiovascular risk (13.2%; n = 22) than participants in Q2 (17.7%, n = 31), Q3 (34.3%; n = 59), and Q4 (49.4%; n = 78). Statistical analysis showed a significant difference in the comparisons between Q4 and Q3 with the other quartiles.

## Discussion

Hyperuricemia has been associated with an increased risk of hypertension, obesity, and cardiovascular disease^19–21^. These disorders are commonly observed in women during the climacteric period due to the loss of estrogen's protective effect^1^. Estrogen promotes efficient renal urate clearance in premenopausal women. After menopause, this clearance is less effective, leading to increased urate levels. Postmenopausal women tend to have higher UA concentrations than premenopausal women, as reported in several studies^22,23^. In addition, a study reported an increase in UA levels in women undergoing both natural and surgical menopause^22^. Given the association between hyperuricemia and cardiovascular risk in climacteric women, we investigated the relationship between serum UA levels and anthropometric and biochemical variables in this population.

Analyses of anthropometric and biochemical variables showed progressively worse results from the first quartile of UA to the fourth. Women in the third and fourth quartiles of UA (> 4.8 mg/dl) had significantly higher median values in all the anthropometric parameters compared to participants in the first and second quartiles (≤ 4.8 mg/dl). In addition to changes in body composition typically seen during the climacteric period, studies have shown associations between UA and different adiposity markers, such as WC, weight, BMI, and BF^24,25^. UA can affect adipocytes by increasing monocyte chemoattractant protein, pro-inflammatory adipocytokines, and cytokine‐like factors, such as tumor necrosis factor α and interleukin-6. These processes can lead to hypoxia, apoptosis, and necrosis of adipocytes. Moreover, UA can reduce adiponectin production, contributing to accelerated lipogenesis and chronic low-grade inflammation^26^. Mouse models have shown that adipose tissue can also produce UA^27^. This can create a cycle, in which visceral fat increases UA levels, and hyperuricemia is associated with higher weight and BMI. In a study of 271 postmenopausal women, UA levels ≥ 4 mg/dl were associated with overweight, hyperglycemia, and hypertriglyceridemia^28^.

Our results indicate that women with UA > 4.8 mg/dl had significantly higher levels of FBG, insulin, and HOMA-IR levels and used more hypoglycemic medications than those with lower urate levels. Adipose tissue, which is a central endocrine organ, plays critical metabolic functions in energy homeostasis and glucose regulation, with both visceral and subcutaneous fat being important contributors^29^. UA induces oxidative stress in adipocytes and reduces adiponectin levels, which are related to insulin resistance^26^. Furthermore, UA can stimulate gluconeogenesis by blocking AMP-activated protein kinase^30,31^. Our findings are consistent with a study by Grygiel-Górniak and et al., which showed a strong association between increased UA concentrations and insulin resistance in postmenopausal women^28^. Additional studies have demonstrated an association between UA and type 2 diabetes mellitus^32,33^.

Concerning the lipid profile, we observed a marked increase in serum concentrations of TG and non-HDL and a significant decrease in HDL levels across the four UA quartiles. Hyperuricemia (UA > 6.0 mg/dl) can induce oxidative stress and generate free radicals, which are associated with dyslipidemia and an increased risk of cardiovascular disease^34,35^. Some studies suggest that TG synthesis requires NADPH, which could lead to increased UA production^26^. Our findings are consistent with previous studies that showed the relationship between serum UA and dyslipidemia in adults^36^. Dobrzyńska and Przysławski showed that postmenopausal women with UA levels ≥ 5.0 mg/dl had significantly elevated values of serum TG, WHtR, and diastolic blood pressure than women with a UA concentration < 5 mg/dl^37^.

As people age, the kidneys gradually filter solutes less effectively, resulting in the retention of substances like urea, creatinine, and UA^38^. In the current study, we found that women classified in Q3 and Q4 (UA > 4.8 mg/dl) had higher serum concentrations of urea and creatinine than those classified in Q1 and Q2 (UA ≤ 4.8 mg/dl). These findings are consistent with a recent study that found higher UA quartiles were associated with elevated levels of urea and creatinine in adults aged between 20 and 93 years old^39^. In addition, another study showed that increased urea levels were associated with a 2.5 times greater likelihood of elevated UA levels^40^.

In this study, we found that climacteric women with UA levels greater than 4.8 mg/dl had a significantly higher frequency of metabolic syndrome and a higher risk of developing the syndrome. Previous research has indicated that high serum UA concentration can negatively influence all metabolic syndrome factors, including central obesity, insulin resistance, high blood pressure, hypertriglyceridemia, and reduced HDL. Upon entering cells via anion carriers, UA causes oxidative stress in vascular smooth muscle cells, endothelial cells, adipocytes, islet cells, renal tubular cells, and hepatocytes. This increases the risk of hepatic fat accumulation and metabolic syndrome^41,42^. Tao et al. reported that the UA/creatinine ratio is strongly associated with metabolic syndrome risk in postmenopausal Chinese women^43^. Other studies also showed a significant association between hyperuricemia and metabolic syndrome in climacteric women^44,45^.

According to our study, blood pressure increased progressively with increasing UA levels. We observed a significant difference between the first and fourth quartiles for both systolic and diastolic blood pressure. We also found a significantly increased cardiovascular risk in participants with higher serum concentrations of UA and a higher frequency of antihypertensive medications use among women with higher serum concentrations of UA.

Clinical, epidemiological, and experimental studies support an association between increased UA levels and hypertension and cardiovascular disease risk^4^. Intracellular urate increase is suggested to be a key factor in primary hypertension pathogenesis^46^. One relevant and widely accepted pathophysiological mechanism by which UA promotes cardiovascular disease is the reduction in levels of nitric oxide (NO). UA reacts with NO in a quick and irreversible reaction, leading to the production of 6-amino-uracil and consequent NO depletion^4^. NO depletion is a major cause of endothelial dysfunction related to hyperuricemia because it controls vascular tone, prevents platelet adhesion and aggregation, and reduces intima proliferation^4,47^. Additionally, UA has been shown to increase angiotensin II expression in vascular endothelial cells and activate the intrarenal renin-angiotensin system, exacerbating endothelial dysfunction^42^. A recent meta-analysis demonstrated an association between high serum urate and elevated blood pressure, which may contribute to cardiovascular disease^48^. Furthermore, other studies have reported UA as a risk factor for hypertension and cardiovascular disease in postmenopausal women^37,49,50^.

The normal reference range for serum UA in women is 1.5 to 6.0 mg/dl^8^. This range was established based on UA levels in individuals without clinical evidence of gout^51,52^. However, it is well established that UA is central to several metabolic processes beyond articular issues^11^. The worldwide increase in circulating UA highlights the need to re-evaluate this reference range^51^. In this study, we analyzed all data according to the UA quartiles. We found that Q1, Q2, and Q3 had lower UA levels than the hyperuricemia cut-off. Furthermore, our data suggested an association between serum UA concentrations greater than 4.8 mg/dl (Q3 and Q4) and impaired metabolic parameters in climacteric women. Malorbeti et al. reported a cut-off of 4.7 mg/dl for predicting total mortality, 5.6 mg/dl for cardiovascular mortality, and 5.7 mg/dl for myocardial infarction-related mortality in a sample of 23,475 subjects. These findings suggest that climacteric women should aim to manage their UA levels, and further studies should review the UA cut-off in this population.

Our study has some limitations that should be acknowledged. Firstly, the cross-sectional design of the study precludes the establishment of a causal relationship between UA levels and metabolic changes. Secondly, although we did not find any significant differences between the volunteers who used diuretic agents and those who did not (data not shown), it is important to note that this medication can raise UA levels, which may have affected our results. Thirdly, we evaluated women in all three stages of reproductive aging. Although each stage tends to present specific changes, all the women included in our study were in the climacteric period and were experiencing the effects of reproductive aging to some extent. Fourthly, unmeasured confounding factors, such as age, smoking, and alcohol consumption, could have potentially biased our results.

In this study, we chose to analyze the data using quartiles instead of using the reference value for hyperuricemia (UA > 6 mg/dl) in order to investigate the association between UA concentrations and metabolic disorders in climacteric women more comprehensively. Our findings suggest that the current cut-off point for hyperuricemia may not be appropriate for this population, as many women with UA levels below 6 mg/dl showed significant alterations. Women with UA levels greater than 4.8 mg/dl should be closely monitored, highlighting the importance of lifestyle changes, such as improvements in diet and physical activity and urate-lowering therapy in specific cases. We recommend conducting further longitudinal studies to confirm our findings, especially since UA is a modifiable risk factor that can be easily assessed during routine clinical visits.

## Conclusion

We have found that higher serum UA concentrations are associated with unfavorable metabolic and clinical parameters in climacteric women. These results suggest that UA may serve as an accurate marker of metabolic risk in the female population. Further studies are needed to establish an ideal cut-off point and clarify the causal relationship between UA levels and metabolic changes in climacteric women.

## Data Availability

The datasets used and/or analyzed during the current study are available from the corresponding author on reasonable request.
